# Unpacking assisted admissions under the *Mental Health Care Act* 17 of 2002

**DOI:** 10.4102/safp.v64i1.5517

**Published:** 2022-04-20

**Authors:** Kalpesh Narsi

**Affiliations:** 1 Discipline of Psychiatry, School of Clinical Medicine, University of KwaZulu-Natal, Durban, South Africa

**Keywords:** assisted, admission, psychiatry, mental health, mental healthcare user, human rights, legislation, *Mental Health Care Act* 17

## Abstract

The *Mental Health Care Act* 17 of 2002 (MHCA) is a progressive piece of mental health legislation that has the potential to transform mental health services by emphasising patient rights and promoting integration and accessibility. The MHCA allows for the care, treatment and rehabilitation of mental healthcare users, who lack the competence to consent but who do not refuse treatment to be managed as assisted users. This article unpacks the concept and procedure of assisted admissions, comparing it with other types of admissions described in the MHCA. Relevant clinical and legal factors influencing the assisted admission status are discussed. The article concludes with a description of the advantages and challenges of assisted care, together with recommendations for its implementation.

## Introduction

Promulgation of the *Mental Health Care Act* 17 of 2002 (MHCA), represented a watershed moment for patient rights in South Africa. Whilst previous mental health legislation focused on ‘certifying’ patients away to distant psychiatric institutes with little recourse, the MHCA emphasises a more human rights-based approach and fosters the integrated care of mental healthcare users (hereafter referred to as ‘users’) at all levels of healthcare services.^1^

The admission status of users is predicated on two conditions: competence and their willingness to receive treatment.^2^ Competence to consent may be defined as the capacity of users to be aware of their rights, options available to them, and the ability to make a relatively consistent choice, which is situation- and time-specific and is voluntary (free from coercion).

Users who are competent to consent and submit voluntarily for treatment are classified as voluntary. Users who are deemed to lack capacity to consent but do not object to care, treatment or rehabilitation (i.e. are cooperative) are classified as assisted, whereas those objecting to or refusing treatment (i.e. are uncooperative) are classified as involuntary users. A fourth type of admission, emergency admission, allows for users incapable of or unable to make an informed decision to be admitted for 24 h, after which they need to be reclassified as voluntary, assisted, involuntary or discharged, with the appropriate process. Table 1 illustrates the similarities and differences between the three types of admissions.

**TABLE 1:** Similarities and differences between voluntary, assisted, and involuntary care as described by the *Mental Health Care Act 17 of 2002* (MHCA).

Key features	Voluntary care	Assisted care	Involuntary care
Capacity to consent	Present	Impaired	Impaired
Cooperation	Willing to receive treatment	Does not refuse treatment	Refuses treatment
MHCA Admission Forms	None	MHCA-4, MHCA-05 (x2), MHCA-07	MHCA-4, MHCA-05 (x2), MHCA-07
72-h observation	Not required	Not required	Required
Forms on completion of 72-h observation	Not relevant	Not relevant	MHCA-06 (x2), MHCA-08 or MHCA-09, MHCA-11 (if transferred)
Informing Mental Health Review Board (MHRB)	Do not need to inform	Forms to be sent within 7 days	Forms to be sent within 7 days
Judicial Review	Not required	Not required	Forms are sent by MHRB to High Court, High Court authorises involuntary treatment
Treating facility	May be treated at any health facility	May be treated at any designated health facility (district or regional hospital)	Must be transferred to a psychiatric hospital if remains involuntary after 72-h observation
Continued and chronic care under assisted/involuntary status	No relevant	Require periodical reports at 6-months and then annually	Require periodical reports at 6-months and then annually
Objection to Treatment	User may exercise right to refuse treatment	Appeal process to be followed	Appeal process to be followed

*Source:* Adapted from *Mental Health Care Act* 17 of 2002, Department of Health, Republic of South Africa.

MHCA, *Mental Health Care Act.*

## Procedure for assisted admissions

Whilst the admission procedure for assisted users initially mirrors the administrative process for involuntary admissions, it is considerably less authoritarian and formal. As assent is obtained from an individual who is not clinically or legally able to consent, assisted treatment may be considered as ‘a third party voluntary procedure’ or consent by proxy.^2^

**FIGURE 1:**
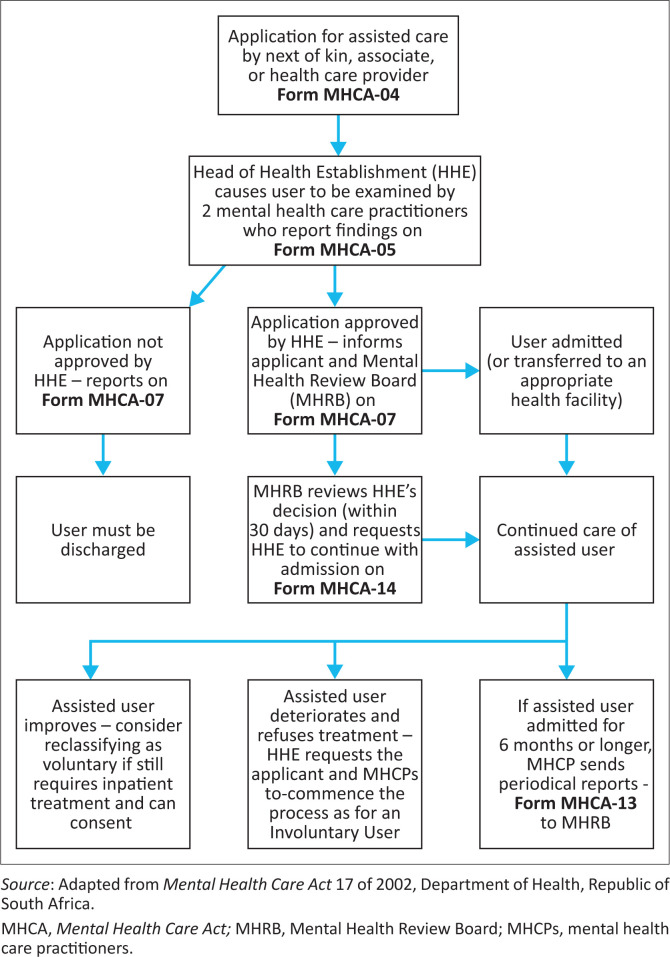
Procedure for assisted admission under the *Mental Health Care Act*.

Sections 26–31 of the MHCA describe the process for assisted care,^3^ which is summarised in Figure 1 and outlined below:

- An application using form MHCA-04 is made by a user’s spouse, next of kin, partner, associate, parent or guardian, provided the applicant is over the age of 18 years (an associate is a person who is in substantial contact with the user or has a substantial or material interest in their well being).
- Where the applicant is less than 18 years, or if the next of kin is unavailable, incapable or unwilling to make an application, a healthcare provider may do so. In such a case, the healthcare provider must record his or her reason for making the application (e.g. ‘next of kin is unavailable’ or ‘relative accompanying the user is 17 years old’) and report steps taken to locate the next of kin (e.g. ‘no contact details are available; a social worker consult will be performed to trace family’ or ‘user’s mother contacted. She is too ill to come to hospital but assented telephonically to the user’s admission’).
- The applicant must have seen the user within 7 days of making the application. The allowance makes it possible to address the logistics of transport or bed availability in resource limited settings, where the applicant might not be able to be physically present at the time of admission.
- The application must be made under oath. In a hospital setting, this is performed in the presence of a commissioner ex officio delegated by the head of the establishment; in the community, this may be the South African Police, or any other entity as stipulated in the Justices of the *Peace and Commissioners of Oaths Act* 16 of 1963.^4^
- On receipt of an application (form MHCA-04), the head of the health establishment (HHE) must have the user assessed by two mental healthcare practitioners (MHCPs), one of whom must be a medical practitioner. The second MHCP may be another medical practitioner, a clinical psychologist, social worker, or nurse or occupational therapist with psychiatric training. If the next of kin was unavailable, and an MHCP filled in an application, he or she cannot be the examining practitioner.
- The MHCPs assess the user and submit their independent written findings on MHCA-05.
- Thereafter, the HHE (or their designee) approves the admission, provided the findings of two MHCPs concur that the reasons for assisted admission are valid, and the HHE is satisfied that the restrictions or intrusions on the user’s rights are necessary to provide them with treatment. The HHE provides notice of this decision on MHCA-07 to the local Mental Health Review Board (MHRB) and the applicant.
- On receiving MHCA-07, the MHRB has 30 days to investigate the merits of the application and authorises the admission on a MHCA-14 form. This usually marks the end of the administrative process of most assisted admission. Unlike for involuntary admissions, there is no 72-hour assessment and no judicial review by High Court.

## Improvement in assisted users’ condition during admission

As soon as a user has clinically improved such that they are able to make informed decisions, they must be converted to a voluntary status should there still be an indication to continue with the admission. As there are no MHCA forms to record this process, it is vital that the treating clinician records this information and decision in the clinical notes. All rights, procedures and processes in the *National Health Act* are applicable to voluntary users (this includes participation in clinical decision-making, informed consent and the right to refuse hospital treatment).^5^ However, if the user is well enough to be discharged back into the community, this is carried out on form MHCA-03. There is no clear legal provision to convert ‘assisted inpatients’ to ‘assisted outpatients’ like for involuntary users, despite the clinical reality that some users, even after an admission, may chronically lack the capacity to consent because of developmental or cognitive impairment.

## Deterioration in assisted users’ condition during admission

When an assisted user, subsequently during his or her admission refuses treatment (i.e. fulfills the condition for an involuntary user), the HHE must advise the applicant (who applied for the assisted admission) and the relevant treating clinicians to make an application for involuntary care. This entails that the legal admission process and the relevant forms be completed *de novo*. However, the MHCA provides a leeway of 30 days to reapply for involuntary admission and the clinical care may continue as is appropriate and necessary. Whilst some MHCA may invariably improve during this time, there are no published local data to inform on the frequency and outcomes regarding this scenario in clinical practice.

## Refusal of hospital treatment and appeals by assisted users

Users or their next of kin may appeal against the decision of the HHE to the MHRB, which must, within 30 days of receipt, evaluate the merits of the appeal based on written or oral representations from the relevant stakeholders (the user, next of kin, appellant, MHCPs and HHE). The process of reclassifying a ‘treatment-refusing assisted user’ to an involuntary user (as described here) must also be halted, pending the investigation by the MHRB. If the MHRB upholds an appeal, all care and treatment must be stopped according to the relevant clinical practice, and the user must be discharged. It is therefore important to note that there is no ‘refusal of hospital treatment’ nor a simple signing out of the user (including by the very next of kin that applied for the assisted admission). This ensures that the rights and provision of clinical care of users are not compromised should the next of kin be averse to and obstructs access to treatment.

## Leave of absence for assisted users

An assisted user may be granted leave of absence (‘pass out’) during his or her admission for up to two months. The terms and conditions of such leave must be stipulated to the user and their custodian. These should be discussed collaboratively and agreed upon, and may typically include adherence to prescribed medication regimes, sobriety from illicit substances and avoidance of activities deemed to place the user’s mental or physical health at risk. Leave of absence may be revoked should the clinical team have reason to believe that the user has not complied with the terms and conditions.

Besides offering users a welcomed reprieve from hospitalisation, leave of absence may be used to assess recovery before discharge.^6^ Social interactions and behavioural problems, often masked by a sterile clinical environment, may surface during the leave. It is therefore prudent to obtain an account of the user’s condition from the custodian so that residual symptoms and challenges with social reintegration may be assessed and therapeutically targeted.

## Consent to medical treatment by assisted users

The assisted (or involuntary) status only applies to the care, treatment and rehabilitation of the mental illness that is focus of intervention for which an application was made. It does not legally confer a ‘blanket’ incapacity to consent. Should a user be able to appreciate and deliberate on the need for medical or surgical treatment of another medical condition, he or she must be afforded the opportunity to provide informed consent for that treatment or procedure, notwithstanding their MHCA status. However, should they lack capacity, then their next of kin may provide such consent. The HHE only provides consent if the user’s next of kin is unavailable and untraceable, and if both the HHE and treating clinician are of the opinion that such treatment is appropriate and recommended after considering relevant alternatives.

## Advantages and challenges in classifying users as assisted

Admitting patients as assisted users has numerous advantages. Assisted users can be managed at district hospitals, which are closer to users’ homes and communities, preventing the stigma and shame that may arise from admission to psychiatric institutions.^1^ Assisted admissions at localised and lower service platforms allow for more integrated care and collaboration with users’ support structures. Furthermore, the admission process is shorter, with fewer forms to fill; there is no requirement for 72-hour observations and no judicial review. Such admissions therefore actualise the MHCA principle of users being managed in the least restrictive manner by acknowledging that users do have opinions on their treatment, and a collaborative approach may be strived for despite their impairment. A systematic review found that whilst staff commonly felt that unconsented treatment compromised positive therapeutic relationships, providing users with information and engaging them in decision-making had a significant impact on users’ experiences.^7^ This, in turn, promotes adherence and better health outcomes.

However, for this to be effective, district hospitals must be capacitated with infrastructure, human resources (including psychologists, social workers and occupational therapists), availability of appropriate pharmaceutical and therapeutic interventions, and outreach from specialist psychiatrists. Local research continues to find a critical shortage of such resources.^1,8,9^ There are also differences and discrepancies in the implementation of the MHCA based on resources, knowledge, perceptions and attitudes.^10^

## Conclusion

Whilst assisted admission of users has clinical, legal, ethical and logistical advantages, numerous systemic challenges pose barriers to relevant users being classified appropriately. In order for successful implementation of the MHCA there is an urgent need for political will, leadership and adequate funding. Regular training of MHCPs, support from outreach psychiatrists and the establishment of clear referral pathways are strongly recommended.
